# Dietary Intake across Reproductive Life Stages of Women in India: A Cross-Sectional Survey from 4 Districts of India

**DOI:** 10.1155/2020/9549214

**Published:** 2020-06-23

**Authors:** Shantanu Sharma, Faiyaz Akhtar, Rajesh Kumar Singh, Sunil Mehra

**Affiliations:** MAMTA Health Institute for Mother and Child, Delhi, India

## Abstract

Nutritional deficiencies among women of reproductive age, especially from socially backward classes, are widely prevalent in India. The present study aimed to assess the nutrient intakes and analyse their associations with sociodemographic attributes among socially backward adolescent girls, newly married women, pregnant women, and lactating mothers from four districts of India. Further, the study looked at the associations between nutrient intakes and anthropometric measurements (body mass index, BMI; waist circumference; and waist-hip ratio, WHR) among adolescents and newly married women. This community-based cross-sectional study used the 24-hour recall method of the dietary survey to assess the food intake of women and girls. Nonparametric tests of associations between sociodemographic characteristics and the median nutrient intakes were conducted. Expected and observed increments in energy and nutrient intakes of pregnant and lactating women from the base (requirement of an adult woman) were calculated. A total of 477 pregnant women, 455 lactating mothers, 532 newly married women, and 223 adolescent girls were interviewed. According to the 24-h dietary recall, only 35% of adolescent girls, 57% newly married women, 40% pregnant women, and 34% lactating mothers were able to meet 70% of the recommended energy requirements. A large percentage of pregnant women had less than 50% of the recommended intakes of iron, calcium, and folic acid. Women living in nuclear families, urban slums, and those from backward classes had lower intakes of almost all the nutrients compared to their counterparts (*p* < 0.001). There were no significant differences in the nutrient intakes of adolescents, newly married, pregnant, and lactating women, and all had poor dietary intakes. We found positive relationships of all three anthropometric measurements (BMI, waist circumference, and WHR) with fats and inverse associations with carbohydrates. Public health interventions should work towards improving the nutrition of these vulnerable populations.

## 1. Introduction

The dropping nutritional status of women across different phases of life, especially from preconception to adolescence, is a cause of concern and focus of the public health research in low- and middle-income countries [1]. Nutritional deficiencies among women of reproductive age (WRA) have transgenerational consequences, as inadequate maternal nutrition is associated with adverse birth outcomes, poor perinatal survival, and altered developmental programming in offsprings [2]. Encased within the vulnerabilities of undernutrition, anemia, inadequate dietary intake, and social status, WRA demand investments on nutrition-sensitive and specific interventions, research, and development [3, 4]. Nutrition during pregnancy and lactation has been the conventional domain of interest, but with the advent of the life-course epidemiology concept, preconception nutrition has gained significance [5]. Hence, the intergenerational approach to nutritional surveillance stances great potential for generating evidence for better programming decisions and monitoring of life stage intervention outcomes.

WRA constitute 55% of the female population and one-fourth of the total population in India [6]. Energy and protein shortages with micronutrient deficiencies of public health importance in the diet are widely prevalent among this group [3]. Interregional, linguistic, cultural, geographical, and food-habiting differences mount substantial influence on dietary behaviours in India. Women from socially and economically backward sections of the society are even more affected compared to their counterparts [7]. Given the limited coverage and longer periodicity of national nutritional surveillance in the country, ad hoc surveys offer a swift dietary evaluation of targeted populations.

There is greater recognition of the effect of dietary intake, not limited to micronutrient deficiencies, on health outcomes and nutritional status of an individual [8]. Research indicates that poor dietary intake concomitant with poor quality and unhealthy dietary patterns impacts body mass index (BMI) at different life stages of women [8–10]. However, limited studies investigating the relationships of dietary intake with BMI, waist circumference, and waist-hip ratio (WHR) found inconsistent results [11, 12]. It is also considered that the effect of diet on BMI, waist circumference, and WHR is mediated by sociodemographic factors, such as age, ethnicity, and lifestyle factors [13].

Moreover, there is a dearth of dietary data in low- and middle- income countries, and the availability of robust data for evidence-based approaches to improve the nutritional status of WRA is a must [14, 15]. Hence, the present study aimed to assess the nutrient intakes and analyse their associations with sociodemographic attributes among socially backward adolescent girls, newly married women, pregnant women, and lactating mothers from four districts of India. This study also aimed to analyse the changing trends of dietary variables across generations from adolescent girls to lactating women. Further, the study looked at the associations between nutrient intakes and anthropometric measurements (body mass index, BMI; waist circumference; and waist-hip ratio, WHR) among adolescents and newly married women.

## 2. Material and Methods

### 2.1. Study Setting

This community-based cross-sectional study, conducted across four regions in India, employed multistage random sampling. The study was a part of the implementation science in project *JAGRITI (means awakening)*. The areas included one state from each region of modern India-Delhi, Karnataka, Bihar, and Rajasthan from the North, South, East, and West regions, respectively. One district per state and one block per district were selected randomly for the survey. So, the four selected districts were West Delhi, Bangalore, Patna, and Sri Ganganagar. Further, ten villages were selected randomly from each block with preference to those with a higher proportion of socially backward sections, including scheduled castes, scheduled tribes, and other backward classes (data extracted from Census 2011). For each village, the number of households selected was proportional to the ratio of the marginalised population. The first household was selected randomly in each area, and subsequent households were selected based on the fraction in systematic random sampling. A similar sampling approach was adopted for the selection of households in urban slums.

### 2.2. Sample Size

Using a 50% prevalence of calorie deficiency in each group, at 95% confidence level, 5% absolute error, and 5% drop rate, the sample size was calculated at 1680 (of all the four groups combined). Only women and girls residing in the study area for the past 1 year or more were interviewed. Pregnant and lactating women were eligible to participate in the dietary survey if they were in their second or third trimester of pregnancy, or breastfeeding an infant or young child <24 months of age, respectively.

### 2.3. Data Collection

The research investigation primarily involved four phases: preinvestigation, training, data collection, and analysis. The preinvestigation phase involved the acquisition and standardization of the software for data analysis, framing, pretesting, and finalization of questionnaires, procuring the standardized tool kit, and conducting a preliminary food habit survey using an open-ended questionnaire by the field investigators at each of the study sites. All investigators were appropriately trained to employ suitable dietary tools for gathering requisite data. Simulation exercises were also conducted to keep interviewer bias to the minimum. Data collection was finished within the stipulated time of 2 months, ensuring the timely completion along with the quality assurance of data through random on-site assessment techniques.

### 2.4. Study Tool

The investigators collected information on sociodemographic particulars of women, such as age, residence (rural areas or urban slums), occupation status, social class (scheduled caste, scheduled tribe, and other backward class or general class), family size, type of family (nuclear or joint/extended), education status, cooking practices (cooking fuel and cooking utensils), and receipt of iron-folic acid tablets and supplementary food at Anganwadi Centre (Integrated Child Development Scheme Centres, ICDS,or Mother and Child Care Centres) [16]. Additionally, information about education and occupation of the head of the household and monthly family income was obtained for assessing the socioeconomic status of women using a modified Kuppuswamy scale [17]. Questions to assess the physical activity of participants included the practice of moderate or vigorous physical activities (walking, jogging, swimming, cycling, exercise, dancing, and yoga or none) and frequency of such activities.

Single 24-hour recall method of dietary survey was carried out to assess the food intake of women and girls. The 24-hour dietary recall interview was divided into five phases: quick list, forgotten foods, time and occasion, detail cycle, and final probe. The different phases were designed to encourage respondents to think about their intake in different ways and from different perspectives. In most of the cases, the respondent herself was the cook and was questioned about the types of food prepared for breakfast, lunch, evening tea, and dinner during the previous day. However, for adolescents, the member of the household who cooked the food was asked about the types of food prepared during the last 24 hours. In addition, information was collected on the cooking practices, such as the type of cooking fuel and utensils. Pregnant women, lactating mothers, and adolescent girls are provided supplementary food at Anganwadi Centres under the ICDS scheme [16]. The supplementary food received from the Anganwadi Centres was included in the 24-hour dietary recall method analysis.

An account of the raw ingredients used in the household for each food preparation was obtained and weighed using standard tool kits provided to the investigators. Different instruments such as kitchen weighing scale, measuring cups, and spoons were used for portion estimation. In the case of inaccessibility to raw ingredients, precalibrated cooking utensils were used to estimate the raw foods. Respondents facing problems in communicating the food items were shown photographs of the local dishes to probe further. Investigators visited Anganwadi Centres for accurate estimation of foods consumed by participants at these places.

To establish the association of dietary intakes with anthropometric measurements, weight, height, waist circumference, and hip circumference of only newly married women and adolescent girls were measured. However, only 205 and 207 adolescent girls gave consent for the measurement of BMI and WHR, respectively. Similarly, only 480 and 484 newly married women provided consent for the measurement for BMI and WHR, respectively. We did not obtain anthropometric measurements of pregnant and lactating women as there is a lack of conclusive evidence on the validity of these parameters when obtained during mid- and late-pregnancy.

Weight was measured to the nearest 0.1 kilograms using an electronic weighing scale with women in light clothing and footwear removed. Height was measured using a standard height metre with the woman in an upright standing position without footwear. Two readings were obtained and an average of the two readings was finally considered. BMI was calculated using the following formula:

(1) $$\begin{array}{c} \text{BMI}=\frac{\text{weight }\left(\text{Kilograms}\right)}{\text{height}{\left(\text{metres}\right)}^{2}}. \end{array}$$

We measured waist circumference around the smallest circumference between the lowest rib and iliac crest. The measurement of the waist circumference was taken at the end of the normal respiration when the woman was standing straight with her arms by her side and feet together. We measured hip circumference horizontally at the level of the greatest lateral extension of the hips. Waist and hip circumferences were measured to the nearest 0.1 cm using an inelastic tape. The WHR was calculated from the waist and hip circumference measurements.

### 2.5. Ethical Considerations

The research study was granted ethical approval by the Institutional Ethical Review Board (MERB/Sep. 2016/003). Participants were informed verbally about the study, its objectives, and the time needed for the same by the investigators. Participants were showed the subject information sheet with details about the study and the contact details of the principal investigator for asking for further information. Written informed consents were obtained from the participants (adults≥18 years). In the case of adolescents (10–17 years), written consents were obtained from parents/guardians, and assents were obtained from adolescents after explaining them about the details of the study. Participants were assured of the confidentiality of their information. No financial or other incentives were offered.

### 2.6. Data Analysis

The entire study was completed in 5 months. Dietary information from the 24-hour recall method was coded, computerized, and computed to derive the daily energy, proteins, and micronutrient intakes. Dietary data were prepared in DietSoft software (based on Indian dietary scenario) for the calculation of nutrients [18]. The software obtained food nutrient values from the Indian food composition table (IFCT), 2017, which contains nutrition composition data for 528 foods [19]. Nutrient values for any food item not included in the DieftSoft were imputed from other sources such as the National Institute of Nutrition database and the United States Department of Agriculture Nutrient Database for Standard Reference, Release 28 [USDA SR28] [20, 21]. Dietary reference intakes (DRI) of the Indian Council of Medical Research (ICMR), 2010, were used for these analyses [22]. Recommended dietary allowances (RDA) were DRI recommendations (Supplementary Table 1). Nutritional adequacy of energy and seven nutrients was assessed (proteins, fats, calcium, vitamin C, iron, zinc, and folic acid).

RDA method was applied to estimate the prevalence of inadequate intakes of energy and nutrients (proteins, fats, vitamin C, iron, folic acid, zinc, and calcium). The prevalence of inadequate intake was categorized into three categories, namely, the proportion of population consuming less than 50% RDA, 50–70% RDA, and >70% RDA of energy and nutrients. The 2010 dietary guidelines by ICMR mentioned that energy recommendations (RDA) for adolescents are based on moderate activity [22]. Percent adequacy of energy and nutrients was calculated for all types of participants by using the following formula:

(2) $$\begin{array}{c} \text{percent adequacy}=\frac{\text{actual intake of the nutrient}}{\text{RDA of that nutrient}}*100. \end{array}$$

An average of the percent adequacies (median) of nutrients for each of these population groups was calculated. This reflected what percentage of the recommended intake was consumed by women and girls in the last 24 hours. Expected and observed increments in energy and nutrient intakes of pregnant and lactating women from the base (requirement of an adult or nonpregnant nonlactating woman, NPNL) were calculated using the following formula:

(3) $$\begin{array}{c} \text{expected increment of nutrients }=\;\frac{\text{RDA for PW or LM}-\text{RDA for NPNL woman}}{\text{RDA for NPNL woman}}*100. \end{array}$$

Similarly,

(4) $$\begin{array}{c} \text{observed increment of nutrients }=\frac{\text{median intake by PW or LM}-\text{median intake by NPNL woman}}{\text{median intake by NPNL woman}}*100, \end{array}$$

where PW is pregnant woman and LM is lactating mother.

For the calculation of observed increments, median intake of nutrients by NPNL women were subtracted from the median intake of nutrients by pregnant women or lactating mothers. In our study, these population groups had different individuals. An observed increment in nutrient intakes was compared to the expected increment. Less than 1% of the findings in energy and nutrient intakes across all categories combined were implausible. Hence, we did not remove them during the analysis.

The data were entered and analysed using IBM SPSS Statistics for Windows version 24.0 (IBM Corp., Armonk, NY, USA). Descriptive statistics were calculated for all the variables. The mean (standard deviation, SD) was used to present normally distributed variables. The median and interquartile range were used to present the average values of energy and all the nutrients due to their skewed distribution. The nonparametric tests (Mann–Whitney test or Kruskal–Wallis test) were used to assess the associations of sociodemographic characteristics and cooking practices with energy and nutrient intakes among women and girls. Median intakes of nutrients were presented along with two-sided *p* values for Mann–Whitney and Kruskal–Wallis tests of significance. Moreover, to establish the associations between dietary intakes and anthropometric measurements (BMI, waist circumference, and WHR as continuous variables), energy-adjusted measures of nutrient intakes (proteins, carbohydrates, and fats) were applied through nutrient density models [23]. Additionally, carbohydrate intake (energy-adjusted) was used for this analysis. Energy-adjusted measures of nutrient intakes were expressed as a percentage of total energy (%E) for proteins, carbohydrates, and total fats. The adolescent girls and newly married women were grouped according to the quintiles of energy-adjusted measures of nutrient intakes (proteins, carbohydrates, and fats).

We used multiple linear regression to establish the differences in average BMI, waist circumference, and WHR between the quintiles of nutrient intakes after adjusting for age, socioeconomic status, social class, category (adolescent or women), and education status. Medians for energy-adjusted nutrient intakes and beta coefficients (95% confidence interval) for linear regression were presented along with two-sided *p* values. The associations with a *p* value <0.05 were considered statistically significant.

## 3. Results

### 3.1. Sociodemographic Characteristics

**Figure 1:**
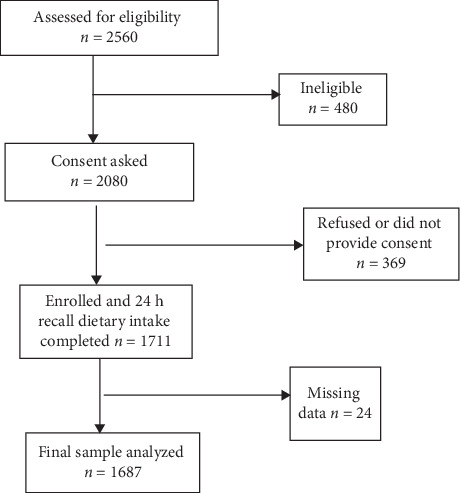
Flowchart of participant progression through the dietary survey across 4 districts.

A total of 477 pregnant women (20–35 years), 455 lactating mothers (20–35 years), 532 newly married nonpregnant and nonlactating women (20–35 years), and 223 adolescent girls (10–19 years) were interviewed in the survey (Figure 1). The characteristics of the study participants are shown in Table 1. The mean (SD) ages of adolescent girls, newly married women, pregnant women, and lactating mothers were 15 (2.3), 23 (3.8), 23 (3.5), and 24 (3.8) years, respectively. Based on the residence, nearly an equal number of participants belonged to rural areas (51.3%) and urban slums (48.7%). The mean (SD) gestational age of pregnant women at the time of the interview was 6.4 (1.5) months. Barring the adolescent group, nearly one-third of participants had studied only till the primary level or lower. Nearly 80% of participants (except the adolescent group) were housewives, and only 10% of the total were employed as unskilled, semiskilled, or skilled workers. Nearly 81% of participants belonged to socially backward classes. More than two-thirds of participants in all the groups belonged to lower socioeconomic status (lower and upper-lower) according to the modified Kuppuswamy scale. Most of the study participants were Hindus (80%), followed by Muslims (7.6%), Jains (7.1%), Sikhs (3.3%), and Christians (2%). The median family size was five members.

### 3.2. Status of Dietary Intakes of Energy and Nutrients

Less than one-fifth of women and girls responded to the question of physical activity. Further, more than 80% of women were housewives. As a result, physical activity was not used for assessing RDA requirements. The intakes of women were compared with the standard requirements of a woman having a sedentary lifestyle (to avoid underreported intakes). The status of dietary intakes of energy and other major nutrients of the study participants are shown in Table 2. Adolescents and newly married women on an average consumed less than three-fourths of the recommended intakes of energy, iron, calcium, folic acid, protein, and zinc in one day. On the contrary, the consumption of fat was 140% of the recommended intake among newly married women. Among all the groups, the intakes (percentage of the recommended intake) of iron, folic acid, protein, and zinc were lowest in pregnant women. Lactating mothers had the least proportion of the recommended intake of calcium compared to the other groups. The expected increment in nutrient intakes of pregnant women and lactating mothers compared to an adult woman has been represented as a percent increase in the RDA (Table 3). The observed increment among pregnant and lactating women in the intakes of energy and all the nutrients were abysmally poor. In fact, the consumption of energy, zinc, and folic acid had decreased instead.

### 3.3. Dietary Intakes across Sociodemographic Groups

Median daily intakes of energy and nutrients among different sociodemographic groups are presented in Table 4. Women residing in urban slums had a significantly lower intake of nearly all the nutrients compared to their counterparts from rural areas (*p* < 0.001). The consumption of energy, proteins, zinc, and vitamin C was the highest among women from Bangalore compared to women from other districts (*p* < 0.001). The iron and vitamin C intakes were the least among residents of Patna. The median intakes of proteins, fats, iron, folic acid, and calcium were maximum among women and girls with the highest education status (*p* < 0.05). Except for energy, zinc, and vitamin C, intakes of all other nutrients were higher among women who were working as skilled labour (*p* < 0.05) than others. The median intakes of energy and most of the nutrients except fats were lower among women from socially backward classes (scheduled castes, tribes, and other backward classes) than from general class (*p* < 0.001). Participants from the upper socioeconomic status had a higher consumption of almost all the nutrients (*p* < 0.001). Also, women living in nuclear families had a lower intake of almost all nutrients in comparison to those from joint/extended families (*p* < 0.001). The consumption of proteins, fats, iron, folic acid, and calcium was more among women and girls registered at Anganwadi Centre compared to those not registered (*p* < 0.05).

As shown in Table 5, only 35% of adolescent girls, 57% newly married women, 40% pregnant women, and 34% lactating women were able to meet 70% of the recommended energy requirements. Around 30% of adolescent girls and lactating mothers consumed less than 50% of the recommended intake of energy. The iron and calcium intakes of nearly two-thirds of adolescent girls were below 50% of the recommended intakes. The protein intake of around 40% of lactating mothers and 51% of pregnant women were below 50% of the recommended intake. A large percentage of pregnant women had less than 50% of the recommended intakes of iron, calcium, and folic acid (81%, 77%, and 96%, respectively).

Mean (SD) BMI and WHR of adolescents were 17.8 (2.8) kg/m^2^ and 0.82 (0.08), respectively. Similarly, mean (SD) BMI and WHR of newly married women were 20.9 (3.8) kg/m^2^ and 0.85 (0.09), respectively. Mean (SD) waist circumference of adolescents and newly married women were 65.8 (11.8) and 74.9 (16.8) cm, respectively. Higher carbohydrate intake was associated with lower mean BMI in adolescents and newly married women (Table 6). Compared to those in the 5^th^ quintile, individuals in the 1^st^, 2^nd^, 3^rd^, and 4^th^ quintiles of carbohydrate intake had, on average, a BMI of −0.7, −1.0, −1.1, and −0.2 lower, respectively (*p*_trend_ = 0.01). A similar association was found between carbohydrate intake and WHR. The association of carbohydrate intake with waist circumference followed similar trends as with BMI. Compared to those in the 5^th^ quintile, individuals in the 1^st^, 2^nd^, 3^rd^, and 4^th^ quintiles of carbohydrate intake had, on average, a WHR of −0.03, −0.02, −0.007, and −0.005 lower, respectively (*p*_trend_ = 0.002).

On the contrary, higher fat intake was associated with higher mean BMI. Compared to those in the 5^th^ quintile, individuals in the 1^st^, 2^nd^, 3^rd^, and 4^th^ quintiles of fat intake had, on average, a BMI of 1.2, 0.2, and 0.2 higher and −0.3 lower, respectively (*p*_trend_ = 0.004). Similarly, compared to those in the 5^th^ quintile, individuals in the 1^st^, 2^nd^, 3^rd^, and 4^th^ quintile of fat intake had, on average, a WHR of 0.04, 0.02, 0.03, and 0.002 higher, respectively (*p*_trend_ = 0.001). There was no statistically significant association of proteins with BMI, waist circumference, and WHR.

## 4. Discussion

Good nutrition is a critical component at every stage of life, from preconception through adolescence to adulthood. In our study, we assessed the dietary intakes of women and highlighted the sociodemographic parameters that influence their intakes. The study brings in light the disproportionate consumption of energy and nutrients among women in various life stages. Most of the studies in the past have analysed dietary intakes of women across different stages in isolation. Our study presented the variations in dietary intakes and their determinants through a continuous life cycle approach.

The maintenance of good health requires approaches that recognize the effect of multiple influencers on an individual, including social, cultural, and environmental accessibility [7]. In the current community-based cross-sectional study, intakes of energy and all major nutrients were found to be lower than the recommended intakes across all the study groups. The study suggests that energy and other nutrient intakes vary by sociodemographic groups among participants. The difference in dietary intakes exists between pregnant or lactating women and newly married women but not to the extent of increment needed as specified by dietary guidelines. The increment in the intakes of nutrients during or after pregnancy was not at par with the suggested norms. In fact, the study identified a decrease in the intakes of energy, folic acid, and zinc during pregnancy. These findings are supported by previous literature specifying that there is no difference in nutrient intakes of pregnant and lactating women, and other NPNL women [24, 25]. Pregnancy is a time of profound physiological changes and increased nutrient requirements for foetal growth and development. Inadequate intake of nutrients during this phase may have a substantial impact on pregnancy outcomes and infant's health [26].

The mean intakes of all the micronutrients among pregnant women were quite similar to the intakes reported in two previous studies (*n* = 50 and *n* = 292) from India [24, 27]. The estimates of most of the nutrients in pregnant women and lactating mothers were similar to the ones reported in another study by Gosh-Jerath et al. except for energy, proteins, fats, and calcium [28]. These estimates were comparatively lower in our study. The plausible justification of such low values in our study could be the higher representation of women from socially backward sections (scheduled castes, tribes, and other backward classes). Our values on the intakes of energy, proteins, and calcium except for iron among pregnant women matched with the preintervention values of a randomized control trial from Varanasi under similar settings as it had 80% of the respondents from backward classes [29]. However, the mean (SD) intake of iron in the Varanasi intervention study was reported to be as high as 19 (7) mg in comparison to the median intake of 11 mg in our study. A reported 80% consumption of iron-folic acid tablets by the subjects in the Varanasi intervention trial could be an important factor.

Our finding of higher fat consumption compared to micronutrients was consistent with previous studies in India, which may be partially due to cereal-based Indian diets, contributing substantially to fat intake [30]. Studies from other Asian countries and the western world also report higher fat intake among pregnant and lactating mothers [31, 32]. It is imperative to understand that high fat intake is associated with the risk of preeclampsia in women and congenital heart defects in the offspring [33, 34]. The dietary intake of women during the postnatal phase is equally important, as the intake during pregnancy, which supports the demand associated with lactation and ensures optimal early life nutrition for the infant. Lactating mothers are at risk of micronutrient deficiencies due to inadequate intake and poor diet quality [35].

Dietary intakes of iron, folic acid, and calcium were found to be notably lower than the RDA among pregnant and lactating women. In fact, more than three-fourths of pregnant women had less than 50% of the recommended intakes of iron, fats, and calcium. Similarly, more than three-fourths of lactating mothers had less than 50% of the recommended intakes of calcium and folic acid, and around two-fifths had less than 50% of the recommended intake of iron in their diet. This might be explained by the higher percentage of the Indian population dependent on plant and cereals-based diets [30]. Despite the provision of iron-folic acid tablets under government schemes to pregnant and lactating mothers, factors such as lack of awareness, poor compliance, incorrect intake practices, and inappropriate accessibility act as barriers to its adequate intake [36]. This worsens the situation resulting in poor maternal and child outcomes.

Previous studies have suggested the associations of dietary intakes among women and girls with sociodemographic attributes and the economic status of the family [7, 24, 37]. Women of higher education, occupation, and economic status tend to report higher intakes of energy and nutrients such as proteins, fats, iron, folic acid, and calcium [24]. Our results also suggest that women at the higher socioeconomic and education level had significantly higher intakes of most of the nutrients. The women in the highest occupation profile (skilled labour) had a significantly higher intakes of most of the nutrients compared to their counterparts in unskilled jobs except energy, vitamin C, and zinc. Our study reaffirms the previous findings that women from socially backward sections of the society have lower micronutrient intakes compared to those belonging to the general class [7]. Further, women residing in urban slums reported lower intakes of energy and most of the nutrients, which is in agreement with previous studies from India [24, 38]. This might be due to the fact that people in urban slums experience poor access to healthcare services, overcrowding, and housing, and are likely to have poor health outcomes [39, 40]. Considering the poor intake of most of the nutrients and limited diet quality among lower sociodemographic groups, it is of increasing importance to develop nutrition-sensitive and specific interventions for women and girls. While using the delivery platforms in community- and facility-based settings, for women across life-course, the nutrition interventions had the potential to mitigate the sociodemographic influences on the dietary intake and nutritional status of women [41].

Contrary to evidence from the studies in the past, our study found that women using unhealthy sources of cooking fuel (solid fuel) have a higher median intakes of energy and most of the nutrients [42]. As identified in the study, a higher reported intake among rural population and the increased use of solid fuel by these women could have led to this result. Solid fuels or biomass fuels (cow dung or wood) have been proven to influence negative outcomes on the nutritional status of mother and child [43].

One of the flagship programs of the country, ICDS is aimed at supplementing the diet of adolescent girls and pregnant and lactating women with energy and proteins. Besides this, women and girls are also provided health and nutrition education along with counselling by the ICDS staff [44]. The positive association of dietary intake with the visit to the ICDS Centre has also been reported in other studies [45, 46]. However, the challenges of the inequitable pattern of use of services, constraints of funding, lack of community awareness, and weak performance of service implementation limit the far greater reach of Anganwadi services [47].

Energy and nutrient intakes were inadequate among adolescent girls and more than 70% of girls had less than 50% recommended intake of iron and calcium. The present study results are consistent with those of other studies reporting decreased nutrient and energy intakes among adolescents [40, 48]. The increased nutritional requirements during adolescence, if not met, can result in transgenerational malnutrition and poor maternal health outcomes in the long run.

Similarly, newly married women, though reported fairly better intakes compared to pregnant women and lactating mothers, had inadequate consumption of most of the nutrients except fats. Studies have reported poor dietary intakes among WRA [49, 50]. The intake of micronutrients, such as folic acid, is indeed crucial during the preconception period, and its supplementation is the most cost-effective approach towards the prevention of neural tube defects among offsprings [51].

In the adjusted analysis, a higher carbohydrate intake was significantly associated with lower BMI in the present study. Contrary to our study, another study from Uganda reported the association between higher intake of carbohydrates and higher BMI [23]. However, substantial evidence revealed an inverse relationship of carbohydrate intake with BMI and waist circumference, similar to our study results [52]. Our results support the growing evidence of the effect of fat intake on BMI and waist circumference [53, 54]. A positive association was found between fat and WHR, whereas an inverse association was found between carbohydrates and WHR in our study. Similar findings are reported in a study from Punjab [55]. High intake of fat, particularly trans fats, has adverse health consequences. High carbohydrate and fat intakes are established risk factors for obesity and cardiovascular diseases, the diseases of public health concern in India [56, 57]. Higher fat intake is common during pregnancy and postpartum period due to local customs and beliefs in India [58]. Studies have shown that maternal over nutrition with “high fat” or “high sugar” diet predisposes the progeny to obesity and metabolic diseases later in life [59]. To the best of the authors' knowledge, their study is one of the few studies from India that assessed the association between nutrient intakes and WHR.

### 4.1. Limitations of the Study

The 24-hour dietary recall method used in the study has its limitations, including recall bias, cost ineffectiveness, and time consumption, among others [60]. The study was limited to the calculation of selected nutrients leaving other crucial ones playing a significant role during pregnancy and lactation like selenium, sodium, vitamins B and E, etc. The findings might not be generalized to all the populations due to higher representation from socially background people in the study. Nutrition markers, such as haemoglobin levels, body mass index, or mid-upper arm circumference, are more effective tools than dietary surveys to understand the nutrition epidemiology of the population. However, due to the limitation of time and monetary resources, these were not used.

## 5. Conclusions

This study concludes that there was no nutritional increment in the diets of pregnant and lactating mothers in comparison to nonpregnant, nonlactating women. Consumption of micronutrients like iron, calcium, and folic acid was the lowest despite free supply of their tablets under various national health programs. Therefore, public health interventions should consider working towards addressing these gaps for reaching the most marginalised (socially and economically backward) populations. Such evidence-based interventions include provision of iron-folic acid and calcium supplementation, supplementary food from Anganwadi Centres, subsidized ration under targeted public health distribution (for poorest of the poor), weight monitoring in pregnancy, and nutrition education [61, 62]. These interventions are already in existence, but systematic weaknesses, logistical gaps, resource scarcity, and poor utilization are common barriers in its effective reach to those in need. There is a need to strengthen integration of services and ensure effective procurement mechanisms for drugs and supplements, training facilities for improved program implementation, and monitoring and evaluation mechanisms.

## Figures and Tables

**Table 1:** Sociodemographic characteristics of study participants (*n* = 1687).

Characteristics	Adolescent girls *n* = 223, *N* (%)	Newly married women *n* = 532, *N* (%)	Pregnant women *n* = 477, *N* (%)	Lactating mothers *n* = 455, *N* (%)	Total *n* = 1687, *N* (%)
Residential area					
Rural	122 (54.7)	272 (51.1)	243 (50.9)	229 (50.3)	866 (51.3)
Urban slums	101 (45.3)	260 (48.9)	234 (49.1)	224 (49.7)	821 (48.7)

District					
Sri Ganganagar	59 (26.5)	170 (32)	138 (28.9)	137 (30.1)	504 (29.9)
Patna	63 (28.3)	102 (19.2)	105 (22)	92 (20.2)	362 (21.5)
West Delhi	72 (32.3)	169 (31.8)	150 (31.4)	149 (32.7)	540 (32)
Bangalore	29 (13)	91 (17.1)	84 (17.6)	77 (16.9)	281 (16.7)

Educational status of women or girls					
Primary or below	24 (10.8)	174 (32.7)	145 (30.4)	139 (30.5)	482 (28.6)
Middle school	99 (44.4)	138 (25.9)	142 (29.8)	165 (36.3)	544 (32.2)
High school	99 (44.4)	170 (32.0)	149 (31.2)	111 (24.4)	529 (31.4)
College and above	1 (0.4)	50 (9.4)	41 (8.6)	40 (8.8)	132 (7.8)

Occupational status of women or girls^*∗*^					
Unskilled work	22 (9.9)	55 (10.3)	26 (5.5)	21 (4.6)	124 (7.4)
Semiskilled work	2 (0.9)	4 (0.8)	4 (0.8)	0 (0.0)	10 (0.6)
Skilled work	3 (1.3)	20 (3.8)	2 (0.4)	4 (0.9)	29 (1.7)
Housewife	14 (6.3)	442 (83.1)	439 (92)	422 (92.7)	1317 (78.1)
Student	182 (81.6)	11 (2.1)	6 (1.3)	8 (1.8)	207 (12.3)

Social class of family					
Schedule tribe	4 (1.8)	14 (2.6)	12 (2.5)	8 (1.8)	38 (2.3)
Other backward classes	69 (30.9)	150 (28.2)	133 (27.9)	131 (29.8)	483 (28.6)
Schedule caste	100 (44.8)	272 (51.1)	250 (52.4)	228 (50.1)	850 (50.4)
General class	50 (22.4)	96 (18)	82 (17.2)	88 (19.3)	316 (18.7)

Socioeconomic status of women^∗^∗^∗^∗					
Lower	8 (3.6)	13 (2.4)	20 (4.2)	11 (2.4)	52 (3.1)
Upper lower	162 (72.6)	399 (75.0)	348 (73.0)	316 (69.5)	1225 (72.6)
Lower middle	47 (21.1)	113 (21.2)	98 (20.5)	105 (23.1)	363 (21.5)
Upper middle	6 (2.7)	7 (1.3)	11 (2.3)	23 (5.1)	47 (2.8)

Family type					
Nuclear	173 (77.6)	265 (49.8)	232 (48.6)	234 (51.4)	904 (53.6)
Extended or joint	50 (22.4)	267 (50.2)	245 (51.4)	221 (48.6)	783 (46.4)

Registered at Anganwadi (ICDS) centre					
Yes	65 (29.1)	16 (3)	323 (67.7)	279 (61.3)	683 (40.5)
No	158 (70.8)	516 (97)	154 (32.3)	176 (38.7)	1004 (59.5)

Cooking fuel					
Solid (cow dung or firewood)	71 (31.8)	211 (39.7)	182 (38.2)	162 (35.6)	626 (37.1)
Liquid (kerosene)	4 (1.8)	10 (1.9)	9 (1.9)	12 (2.6)	35 (2.1)
Gas (Biogas/LPG)	148 (66.4)	311 (58.5)	286 (60)	281 (61.8)	1026 (60.8)

Cooking utensils					
Iron	11 (4.9)	35 (6.6)	31 (6.5)	29 (6.4)	106 (6.3)
Other metals (copper/aluminium)	107 (48.0)	248 (46.6)	228 (47.8)	221 (48.6)	804 (47.7)
Stainless steel	105 (47.1)	249 (46.8)	218 (45.7)	205 (45.1)	777 (46.1)

Receipt of iron-folic acid tablets					
Yes	49 (21.9)	26 (4.8)	192 (40.2)	124 (27.2)	391 (23.1)
No	32 (14.3)	41 (7.7)	99 (20.7)	159 (34.9)	331 (19.6)
Missing	142 (63.6)	465 (87.4)	186 (38.9)	172 (37.8)	965 (57.2)

Receipt of supplementary food at ICDS					
Received	34 (15.2)	NA	222 (46.5)	213 (46.8)	469 (40.6)
Not received	189 (84.8)	NA	255 (53.5)	242 (53.2)	686 (59.4)

ICDS, Integrated Child Development Service Scheme; LPG, liquefied petroleum gas; NA,ot applicable. ^*∗*^Occupations: unskilled work included maids, servants, gatekeepers, cleaner, helper, sweeper farmer, etc.; semiskilled work includes drivers, waiters, etc.; skilled work includes technicians, electricians, tailors, cooks, etc. ^^∗^∗^^^∗^∗^Significant differences were found using Kruskal–Wallis test in energy and all the nutrients (*p* < 0.001).

**Table 2:** Daily median (IQR) energy and nutrient intakes of the population (*n* = 1687).

Nutrients	Adolescents	% AD	Newly married females	% AD	Pregnant women	% AD	Lactating mothers	% AD	*p* value
Energy (kcal)	1340.5 (1087.41689.5)	61.7	1404.4 (1123.2–1692.5)	73.9	1391.6 (1152.3–1822.8)	61.8	1434.7 (1434.7–1874.6)	59.3	**0.008** ^*∗*^
Protein (g)	35.2 (27.1–43.3)	69.4	37.8 (29.9–46.9)	68.7	38.5 (30.6–47.9)	49.3	39.3 (31.4–51.2)	55.3	**0.00** ^*∗*^
Fat (g)	25.4 (18.8–40.9)	79.3	27.4 (19.0–40.6)	140.0	28.0 (19.0–48.3)	93.3	29.3 (19.7–48.3)	97.6	**0.06**
Calcium (mg)	233.8 (165.9–403.3)	31.2	294.5 (192.4–493.4)	49.1	334.2 (194.8–580.0)	27.8	305.4 (193.1–511.0)	25.5	**0.00** ^*∗*^
Iron (mg)	9.9 (7.3–13.5)	40.0	11.0 (7.6–15.4)	52.3	11.1 (7.6–15.2)	31.7	11.7 (8.3–15.7)	55.7	**0.02** ^*∗*^
Vitamin C (mg)	43.1 (21.7–81.1)	107.5	35.7 (18.0–76.6)	90.0	43.0 (20.8–80.9)	71.6	37.7 (15.6–72.7)	47.5	**0.046** ^*∗*^
Zinc (mg)	5.1 (3.8–6.5)	48.5	5.3 (4.2–6.6)	53.0	5.27 (4.0–6.7)	43.9	5.5 (4.1–6.9)	45.8	0.14
Folic acid total (mcg)	91.2 (63.5–131.5)	53.0	101.9 (66.2–137.8)	51.0	98.5 (68.2–135.7)	25.0	101.3 (73.2–138.9)	33.6	**0.07**

AD, adequacy; g, gram; mg, milligram; mcg, microgram; kcal, kilocalories; IQR : difference in the upper and lower medians. ^*∗*^*p* value < 0.05 is considered statistically significant.

**Table 3:** Expected versus observed increment in energy and nutrients intakes among pregnant women and lactating mothers.

Nutrients	Expected increment (*I*_P_ − *I*_Y_)/*I*_Y_ ∗ 100	Observed increment (pregnant women)	Expected increment (*I*_L_ − *I*_Y_)/*I*_Y_ ∗ 100	Observed increment (lactating mothers)
Energy (kcal)	18.4	−1	27.3	2.1
Protein (g)	42	1.8	29	4
Fat (g)	50	2.1	50	7
Calcium (mg)	100	13.4	100	3.7
Iron (mg)	67	1	0	6.3
Vitamin C (mg)	50	20.4	100	5.6
Zinc (mg)	20	−0.5	10	3.7
Folic acid (mcg)	150	−3.3	50	−0.5

I_P_, intake among pregnant women; I_Y_, intake among adult females; I_L_, intake among lactating mothers; g, gram; mg, milligram; mcg, microgram; kcal, kilocalories.

**Table 4:** Association of sociodemographic characteristics and cooking practices with the median intakes of nutrients among women and girls (*n* = 1687).

Sociodemographic parameters	Energy (kcal)	Fats (g)	Proteins (g)	Iron (mg)	Vitamin C (mg)	Zinc (mg)	Calcium (mg)	Folic acid total (mcg)
Residential area^*β*^								
Rural	1507.4	34.1	34.1	34.1	34.1	34.1	34.1	34.1
Urban slums	1330.3	1330.3	1330.3	1330.3	1330.3	1330.3	1330.3	1330.3

Districts^¶^								
Sri Ganganagar	1324.8	38.3	36.4	12.5	30.5	4.6	500.9	115
Patna	1401	22.7	36.75	5.8	16.1	4.9	209.6	82.2
West Delhi	1292.4	22.2	38.9	12.2	45	5.6	308.3	107.4
Bangalore	1760.7	27.2	39.9	10.4	71	6.4	169	75.9

Educational status of the women or girls^ǁ^								
Primary or below	1438.7	26.6	37.1	11.5	44	5.7	270.8	94
Middle school	1350	29.5	36.5	10.8	35.4	5	319	99.5
High school	1415.8	25.6	39.2	10.6	38.7	5.2	282.4	100.9
College and above	1517.9	32.8	42.7	11.8	35	5.3	415.3	116.2

Occupational status of the women or girls^§^								
Unskilled labour	1291.9	26.9	35.2	11.5	32.6	4.8	318.6	100
Semiskilled work	1164.6	22.8	35.3	11.4	21.8	5	289.6	92
Skilled work	1260.3	30.1	39.6	11.1	26.4	4.2	373.9	122.3
Housewife	1428.2	28.2	38.6	11.3	40.5	5.4	301.4	100.5
Students	1390	25.4	35.5	9.9	43	5.3	243.8	90.9

Social class of the family^∗^∗								
Scheduled tribe	1478.4	22.9	36.1	7.9	27.5	5.4	185.4	84.5
OBC	1496.9	29.3	39.7	10.7	40	5.3	273.8	95
Scheduled caste	1377.9	28.2	36.6	10.8	35.9	5.1	299.7	98.7
General class	1343.5	24	39.6	12.5	47.6	5.7	316	110

Socioeconomic status of women^∗^∗^∗^∗								
Lower	1326.9	27.9	34.6	10.3	26.4	5.1	314.9	94.5
Upper lower	1391.0	28.1	37.2	10.8	37.9	5.2	288.3	97.5
Lower middle	1402.3	25.6	40.1	12.0	44.6	5.5	302.3	101.9
Upper middle	1709.8	41.3	43.9	13.0	58.5	6.2	481.2	118.2
Type of family^a^								
Nuclear	1397.6	1397.6	1397.6	1397.6	1397.6	1397.6	1397.6	1397.6
Extension or joint	1411.2	1411.2	1411.2	1411.2	1411.2	1411.2	1411.2	1411.2

Registered at Anganwadi (ICDS) Centre^¶¶^								
Yes	1391	29.6	39	12	43	5.3	364.4	102
No	1411	26.6	37	10.5	37	5.3	271.6	96

Cooking fuel^§§^								
Solid (cow dung or firewood)	1490	32.3	37.5	11.7	44.4	5.3	338.8	99.7
Liquid (kerosene)	1463	22.6	36	5.7	21.4	4.4	222.8	79.3
Gas (Biogas/LPG)	1368	25.3	38.4	10.7	37.2	5.3	282.4	100

Cooking utensils^ǁǁ^								
Iron	1285	27.2	39.5	13	44.8	5.6	353.3	112.1
Other metals (copper/aluminium)	1535	24.2	39.7	10.4	44.4	5.8	236.5	91.5
Stainless steel	1330	31.1	35.9	11.4	31.9	5	375.6	104.4

ICDS, Integrated Child Development Service Scheme; LPG, liquefied petroleum gas; INR, Indian rupees; g, gram; mg, milligram; mcg, microgram; kcal, kilocalories. Data include medians for nutrient intakes and *p* values for trends. ^a^Significant differences were found using Mann–Whitney *U* test for the nutrients (*p* < 0.001) including fats, calcium, iron, and folic acid. ^*β*^Significant differences were found using Mann–Whitney *U* test for the energy and the nutrients (*p* < 0.001) including fats, calcium, iron, and vitamin C. ^^∗^∗^Significant differences were found using Kruskal–Wallis test in energy and all nutrients (*p* < 0.001). ^§^Significant differences were found using Kruskal–Wallis test in energy and nutrients (*p* < 0.05) including proteins, carbohydrates, and calcium. ^ǁ^Significant differences were found using Kruskal–Wallis test in energy (*p* < 0.05) and all nutrients (*p* < 0.001). ^¶^Significant differences were found using Kruskal–Wallis test in energy and all the nutrients (*p* < 0.001). ^§§^Significant differences were found using Kruskal–Wallis test in energy and all nutrients (*p* < 0.05) except proteins (*p*=0.2). ^ǁǁ^Significant differences were found using Kruskal–Wallis test in energy and all nutrients (*p* < 0.001). ^¶¶^Significant differences were found using Mann–Whitney *U* test for all nutrients (*p* < 0.05) except energy, zinc, and vitamin C. ^^∗^∗^^^∗^∗^Significant differences were found using Kruskal–Wallis test in energy and all the nutrients (*p* < 0.001).

**Table 5:** Energy and nutrients intake as percentage of the recommended dietary allowance (RDA) among women and girls (*n* = 1687).

Nutrients	Adolescent girls *n* = 223, *N* (%)	Newly married females *n* = 532, *N* (%)	Pregnant women *n* = 477, *N* (%)	Lactating mothers *n* = 455, *N* (%)
Energy (kcal)				
<50% RDA	64 (28.7)	63 (11.8)	111 (23.3)	135 (29.7)
50–70% RDA	81 (36.3)	168 (31.6)	174 (36.5)	162 (35.6)
>70% RDA	78 (35)	301 (56.6)	192 (40.3)	158 (34.7)

Protein (g)				
<50% RDA	49 (21.9)	92 (17.3)	243 (50.9)	180 (39.6)
50–70% RDA	72 (32.2)	184 (34.6)	157 (32.9)	155 (34.1)
>70% RDA	102 (45.7)	256 (48.1)	77 (16.1)	120 (26.4)

Fat (g)				
<50% RDA	53 (23.7)	17 (3.2)	64 (13.4)	63 (13.8)
50–70% RDA	49 (21.9)	55 (10.3)	80 (16.8)	67 (14.7)
>70% RDA	121 (54.2)	460 (86.5)	333 (69.8)	325 (71.4)

Iron (mg)				
<50% RDA	157 (70.4)	242 (45.5)	386 (80.9)	185 (40.7)
50–70% RDA	39 (17.5)	138 (25.9)	48 (10.1)	130 (28.6)
>70% RDA	27 (12.1)	152 (28.6)	43 (9)	140 (30.8)

Calcium (mg)				
<50% RDA	162 (72.6)	279 (52.4)	366 (76.7)	370 (81.3)
50–70% RDA	29 (13)	89 (16.7)	56 (11.7)	43 (9.5)
>70% RDA	32 (14.4)	164 (30.8)	55 (11.5)	42 (9.2)

Zinc (mg)				
<50% RDA	127 (56.9)	217 (40.8)	305 (63.9)	273 (60)
50–70% RDA	58 (26)	204 (38.3)	107 (22.4)	118 (25.9)
>70% RDA	38 (17)	111 (20.9)	65 (13.6)	204 (38.3)

Vitamin C (mg)				
<50% RDA	51 (22.9)	152 (28.6)	173 (36.3)	235 (51.6)
50–70% RDA	26 (11.7)	68 (12.8)	58 (12.2)	63 (13.8)
>70% RDA	146 (65.5)	312 (58.6)	246 (51.6)	157 (34.5)

Folic acid (mcg)				
<50% RDA	95 (42.6)	255 (47.9)	458 (96)	354 (77.8)
50–70% RDA	63 (28.2)	148 (27.8)	17 (3.6)	68 (14.9)
>70% RDA	65 (29.2)	129 (24.2)	2 (0.4)	33 (7.3)

g, gram; mg, milligram; mcg, microgram; kcal, kilocalorie.

**Table 6:** Differences in average BMI and WHR according to quintiles of nutrient intake.

Quintiles of nutrient intake						
Nutrients	1 (lowest)	2	3	4	5 (highest)	*p* value
Carbohydrate (%E)	50.702	61.655	69.645	74.516	79.407	
Β^*∗*^	−0.719 (−1.544, 0.107)	−1.097 (−1.912, −0.281)	−1.174 (−1.971, −0.378)	−0.239 (−1.006, 0.529)	Reference	**0.012**
Β^*∗∗*^	−0.038 (−0.059, −0.016)	−0.026 (−0.047, −0.005)	−0.007 (−0.028, 0.014)	−0.005 (−0.025, 0.015)	Reference	**0.002**
B^*∗∗∗*^	−5.613 (−9.087, −2.139)	−5.363 (−8.783, −1.943)	−3.213 (−6.584, 0.157)	−0.537 (−3.811, 2.737)	Reference	**0.001**

Fats (%E)	9.775	13.843	18.361	24.703	34.881	
Β^*∗*^	1.242 (0.384, 2.100)	0.253 (−0.531, 1.037)	0.241 (−0.585, 1.068)	−0.389 (−1.249, 0.472)	Reference	**0.004**
Β^*∗∗*^	0.045 (0.022, 0.067)	0.019 (−0.002, 0.039)	0.033 (0.011, 0.054)	0.002 (−0.020, 0.025)	Reference	**0.001**
B^*∗∗∗*^	6.791 (3.212, 10.371)	3.720 (0.406, 7.033)	2.375 (−1.099, 5.849)	−0.635 (−4.253, 2.984)	Reference	**0.001**

Proteins (%E)	7.7426	9.54	10.792	12.107	14.169	
Β^*∗*^	−0.031 (−0.871, 0.808)	−0.02 (−0.892, 0.844)	−0.092 (−0.933, 0.750)	−0.490 (−1.319, 0.339)	Reference	0.740
Β^*∗∗*^	−0.017 (−0.039, 0.005)	−0.020 (−0.043, 0.003)	−0.020 (−0.042, 0.002)	−0.014 (−0.035, 0.008)	Reference	0.377
B^*∗∗∗*^	−1.492 (−5.036, 2.052)	−2.271 (−5.924, 1.383)	−2.628 (−6.166, 0.909)	−1.782 (−5.277, 1.712)	Reference	0.653

Energy	931.011	1192.400	1402.290	1682.090	2289.200	
Β^*∗*^	−0.867 (−1.705, −0.029)	−0.128 (−0.991, 0.735)	−0.301 (−1.132, 0.531)	−0.204 (−1.064, 0.656)	Reference	0.247
Β^*∗∗*^	−0.02 (−0.04, −0.00)	0.00 (−0.02, −0.02)	−0.01 (−0.04, 0.00)	−0.00 (−002, 0.02)	Reference	0.131
B^*∗∗∗*^	−1.638 (−5.179, 1.903)	−1.500 (−5.146, 2.146)	−2.429 (−5.941, 1.084)	−2.388 (−6.031, 1.254)	Reference	0.684

BMI: body mass index; WHR: waist-hip ratio. Β^*∗*^: beta coefficients for body mass index, Β^*∗∗*^: beta coefficients for waist-hip ratio, and B^*∗∗∗*^: beta coefficients for waist circumference. Data include medians for energy adjusted nutrient intakes, *β* coefficients (95% Confidence Interval) for linear regression, and *p* values for trend. The difference in average BMI and WHR for each quintile of nutrient intake has been adjusted for age, social class, socioeconomic status, category (adolescent and newly married women), and education status.

## Data Availability

The data related to this manuscript is not provided due to institutional policy but can be made available on personal request.

## Supplementary Materials

Supplementary Materials Supplementary Table 1: recommended dietary allowances for Indians (macronutrients and minerals).

## References

[1] Arora N. K., Mohapatra A., Gopalan H. S. (2017). Setting research priorities for maternal, newborn, child health and nutrition in India by engaging experts from 256 indigenous institutions contributing over 4000 research ideas: a CHNRI exercise by ICMR and INCLEN. *Journal of Global Health*.

[2] Padmanabhan V., Cardoso R. C., Puttabyatappa M. (2016). Developmental programming, a pathway to disease. *Endocrinology*.

[3] Meshram, Balakrishna N., Sreeramakrishna K. (2016). Trends in nutritional status and nutrient intakes and correlates of overweight/obesity among rural adult women (≥18–60 years) in India: national Nutrition Monitoring Bureau (NNMB) national surveys. *Public Health Nutrition*.

[4] WHO (2014). *Comprehensive Implementation Plan on Maternal, Infant and Young Child Nutrition*.

[5] Bernstein M., MacMohan K. (2018). Nutrition overview. *Nutrition across Life Stages*.

[6] Census of India (2011). *Population Enumeration Data*.

[7] Navaneetham K., Jose S. (2008). A factsheet on women’s malnutrition in India. *Economic and Political Weekly*.

[8] Harbury C., Collins C. E., Callister R. (2019). Diet quality is lower among adults with a BMI ≥40 kg m^−2^ or a history of weight loss surgery. *Obesity Research & Clinical Practice*.

[9] Parker H. W., Tovar A., McCurdy K., VadivelooM (2019). Associations between prepregnancy BMI, gestational weight gain, and prenatal diet quality in a national sample. *PLoS ONE*.

[10] Gutiérrez-Pliego L. E., Camarillo-Romero E., Montenegro-Morales L. P. (2016). Dietary patterns associated with body mass index (BMI) and lifestyle in Mexican adolescents. *BMC Public Health*.

[11] López-Olmedo N., Popkin B. M., Mendez M. A., Taillie L. S. (2019). The association of overall diet quality with BMI and waist circumference by education level in Mexican men and women. *Public Health Nutrition*.

[12] Asghari G., Mirmiran P., Yuzbashian E., Azizi F. (2017). A systematic review of diet quality indices in relation to obesity. *British Journal of Nutrition*.

[13] Fletcher E. A., Lamb K. E., McNaughton S. A. (2017). Cross-sectional and prospective mediating effects of dietary intake on the relationship between sedentary behaviour and body mass index in adolescents. *BMC Public Health*.

[14] Micha R., Coates J., Leclercq C., Charrondiere U. R., Mozaffarian D. (2018). Global dietary surveillance: data gaps and challenges. *Food and Nutrition Bulletin*.

[15] Wessells K. R., Young R. R., Ferguson E. L., Ouédraogo C. T., Faye M. T., Hess S. Y. (2019). Assessment of dietary intake and nutrient gaps, and development of food-based recommendations, among pregnant and lactating women in zinder, Niger: an optifood linear programming analysis. *Nutrients*.

[16] Saxena N. C., Srivastava N. (2009). ICDS in India: policy, design and delivery issues. *IDS Bulletin*.

[17] Wani R. (2019). Socioeconomic status scales-modified Kuppuswamy and Udai Pareekh’s scale updated for 2019. *Journal of Family Medicine and Primary Care*.

[18] DietSoft. http://dietsoft.in/

[19] Longvah T., Ananthan R., Bhaskarachary K., Venkaiah K. (2017). *Indian Food Composition Tables 2017*.

[20] Gopalan C., Rama Sastri B. V., Balasubramanium S. C. (2012). *Nutritive Value of Indian Foods*.

[21] US Department of Agriculture Research Service, Nutrient Data Laboratory, USDA National Nutrient Database for Standard Reference, 2016

[22] National Institute of Nutrition (2011). *Dietary Guidelines for Indians: A Manual*.

[23] Matovu N., Matovu F. K., Sseguya W., Tushemerirwe F. (2017). Association of dietary intake and BMI among newly diagnosed type 2 diabetes patients attending diabetic clinics in Kampala. *BMC Nutrition*.

[24] Kumar R., Aslesh O., Kumar A. (2013). Urban poor women do not increase their diet during pregnancy: a study from an urban resettlement colony in Delhi, India. *International Journal of Medicine and Public Health*.

[25] National Nutrition Monitoring Bureau (2012). Diet and nutritional status of rural population, prevalence of hypertension and diabetes among adults and infant and young child feeding practices.

[26] Mousa A., Naqash A., Lim S. (2019). Macronutrient and micronutrient intake during pregnancy: an overview of recent evidence. *Nutrients*.

[27] Durrani A., Rani A. (2011). Effect of maternal dietary intake on the weight of the newborn in Aligarh city, India. *Nigerian Medical Journal*.

[28] Ghosh-Jerath S., Devasenapathy N., Singh A., Shankar A., Zodpey S. (2015). Ante natal care (ANC) utilization, dietary practices and nutritional outcomes in pregnant and recently delivered women in urban slums of Delhi, India: an exploratory cross-sectional study. *Reproductive Health*.

[29] Shivalli S., Srivastava R. K., Singh G. P. (2015). Trials of improved practices (TIPs) to enhance the dietary and iron-folate intake during pregnancy-a quasi experimental study among rural pregnant women of Varanasi, India. *PloS One*.

[30] Green R., Milner J., Joy E. J. M., Agrawal S., Dangour A. D. (2016). Dietary patterns in India: a systematic review. *British Journal of Nutrition*.

[31] Yang J., Dang S., Cheng Y. (2017). Dietary intakes and dietary patterns among pregnant women in Northwest China. *Public Health Nutrition*.

[32] Blumfield M. L., Hure A. J., Macdonald-Wicks L., Smith R., Collins C. E. (2012). Systematic review and meta-analysis of energy and macronutrient intakes during pregnancy in developed countries. *Nutrition Reviews*.

[33] Collins R. T., Yang W., Carmichael S. L. (2020). Maternal dietary fat intake and the risk of congenital heart defects in offspring. *Pediatric Research*.

[34] Arvizu M., Afeiche M. C., Hansen S., Halldorsson T. F., Olsen S. F., Chavarro J. E. (2019). Fat intake during pregnancy and risk of preeclampsia: a prospective cohort study in Denmark. *European Journal of Clinical Nutrition*.

[35] Lebrun A., Plante A.-S., Savard C. (2019). Tracking of dietary intake and diet quality from late pregnancy to the postpartum period. *Nutrients*.

[36] Sonkar V. K., Khan N. M., Domple V. K., Inamdar I. F. (2017). Knowledge and practices of pregnant women regarding the iron supplementation during pregnancy. *International Journal of Community Medicine And Public Health*.

[37] Fledderjohann J., Vellakkal S., Stuckler D. (2016). Breastfeeding, pregnant, and non-breastfeeding nor pregnant women’s food consumption: a matched within-household analysis in India. *Sexual & Reproductive Healthcare*.

[38] Chopra H., Chheda P., Kehoe S. (2012). Dietary habits of female urban slum-dwellers in Mumbai. *Indian Journal of Maternal and Child Health: Official Publication of Indian Maternal and Child Health Association*.

[39] Kapadia-Kundu N., Kanitkar T. (2002). Primary healthcare in urban slums. *Economic and Political Weekly*.

[40] Swaminathan H., Mukherji A. (2012). Slums and malnourishment: evidence from women in India. *American Journal of Public Health*.

[41] Fox E. L., Davis C., Downs S. M., Werner S., Fanzo J. (2019). Who is the woman in women’s nutrition? A narrative review of evidence and actions to support women’s nutrition throughout life. *Current Developments in Nutrition*.

[42] Radhika M. S., Swetha B., Kumar B. N., Krishna N. B., Laxmaiah A. (2018). Dietary and nondietary determinants of nutritional status among adolescent girls and adult women in India. *Annals of the New York Academy of Sciences*.

[43] Bhagowalia P., Gupta P. Nutritional status and access to clean fuels: evidence from South Asia.

[44] Sankar R., Briel T. V. D. (2014). Prospects for better nutrition in India. *Asia Pacific Clinical Nutrition Society*.

[45] Agarwal K. N., Agarwal D. K., Agarwal A. (2000). Impact of the integrated child development services (ICDS) on maternal nutrition and birth weight in rural Varanasi. *Indian Pediatrics*.

[46] Chudasama R. K., Kadri A. M., Verma P. B. (2014). Evaluation of integrated child development services program in Gujarat, India. *Indian Pediatrics*.

[47] Chakrabarti S., Raghunathan K., Alderman H., Menon P., Nguyen P. (2019). India’s Integrated Child Development Services programme; equity and extent of coverage in 2006 and 2016. *Bulletin of the World Health Organization*.

[48] Kawade R. (2012). Zinc status and its association with the health of adolescents: a review of studies in India. *Global Health Action*.

[49] Mittal P. C., Kumar D., Dwivedi S. (2010). Socio-demographic correlates of dietary energy intakes in an Indian community. *Indian Journal of Community Medicine: Official Publication of Indian Association of Preventive &amp; Social Medicine*.

[50] Agrawal A., Varma K. (2016). Diet and nutrient intakes in urban women of Rajasthan State, Northern India. *Ecology of Food and Nutrition*.

[51] Gomes S., Lopes C., Pinto E. (2016). Folate and folic acid in the periconceptional period: recommendations from official health organizations in thirty-six countries worldwide and WHO. *Public Health Nutrition*.

[52] Gaeser G. A. (2007). Carbohdrate quantity and quality in relation to body mass index. *Journal of the Academy of Nutrition and Dietetics*.

[53] Hooper L., Abdelhamid A., Moore H. J., Douthwaite W., Skeaff M. C., Summerbell C. D. (2012). Effect of reducing total fat intake on body weight: systematic review and meta-analysis of andomized controlled trials and cohort studies. *BMJ*.

[54] Koh-Banerjee P., Chu N.-F., Spiegelman D. (2003). Prospective study of the association of changes in dietary intake, physical activity, alcohol consumption, and smoking with 9-y gain in waist circumference among 16 587 US men. *The American Journal of Clinical Nutrition*.

[55] Grewal D. K., Bains K., Kaur H. (2018). A comparison of macro and micronutrient intake of adult men with different degrees of abdominal obesity. *Journal of Applied and Natural Science*.

[56] Zamora-Kapoor A., Sinclair K., Nelson L., Lee H., Buchwald D. (2019). Obesity risk factors in american indians and alaska natives: a systematic review. *Public Health*.

[57] Ahirwar R., Mondal P. R. (2019). Prevalence of obesity in India: a systematic review. *Diabetes & Metabolic Syndrome: Clinical Research & Reviews*.

[58] Lee S. E., Talegawkar S. A., Merialdi M., Caulfield L. E. (2013). Dietary intakes of women during pregnancy in low- and middle-income countries. *Public Health Nutrition*.

[59] Mani I., Dwarkanath P., Thomas T., Thomas A., Anura V. (2016). Maternal fat and fatty acid intake and birth outcomes in a South Indian population. *International Journal of Epidemiology*.

[60] Shim J.-S., Oh K., Kim H. C. (2014). Dietary assessment methods in epidemiologic studies. *Epidemiology and Health*.

[61] Ramakrishnan U., Lowe A., Vir S. (2012). Public health interventions, barriers, and opportunities for improving maternal nutrition in India. *Food and Nutrition Bulletin*.

[62] USAID (2017). Addressing barriers to maternal nutrition: evidence and program considerations. https://www.mcsprogram.org/wp-content/uploads/2017/05/MCSPNutritionBriefBarriersToMaternalNutrition-1.pdf.

