# Supplementary material for: Dietary Intake across Reproductive Life Stages of Women in India: A Cross-Sectional Survey from 4 Districts of India
Source: J Nutr Metab. 2020 Jun 23;2020:9549214. doi: 10.1155/2020/9549214 (PMC7341409; doi:10.1155/2020/9549214)
Supplement: Supplementary Materials — Supplementary Table 1: recommended dietary allowances for Indians (macronutrients and minerals). [file 9549214.f1.pdf]

Supplementary Table 1. Recommended Dietary Allowances for Indians (Macronutrients and Minerals)

Target groups	Energy Kcal/d	Visible fat g/d	Protein g/d	Iron mg/d	Calcium mg/d
Pregnant women	2250	30	78	35	1200
Lactating women	2420	30	71	21	1200
Adolescent Girls 10-12 years	2010	35	40.4	27	800
13-15 years	2330	40	51.9	27	800
16-17 years	2440	35	55.5	26	800
18-19 years	1900	20	55	21	800
Adolescent boys 10-12 years	2190	35	39.9	21	800
13-15 years	2750	45	54.3	32	800
16-17 years	3020	50	61.5	28	800
18-19 years	2320	25	60	17	800
Young female	1900	20	55	21	600
Young male	2320	25	60	17	600

*g/d-grams/day; mg/d-milligrams/day; Kcal/d-Kilocalories/day

Source: Dietary guidelines for Indians, Indian Council of Medical Research 2010
